# Narrative Group Interventions to Rediscover Life Wisdom Among Hong Kong Chinese Older Adults: A Waitlist RCT Study

**DOI:** 10.1093/geroni/igaa057.260

**Published:** 2020-12-16

**Authors:** Esther Chow, Sai-Fu Fung

**Affiliations:** 1 City University of Hong Kong, Kowloon, China; 2 City University of Hong Kong, Kowloon Tong, China

## Abstract

Objectives: To recognize, and rejuvenate the life wisdom of Hong Kong Chinese older adults, a new strength- and meaning-based Narrative Therapy (NT) in practice is developed, with two objectives: to examine its effectiveness in enhancing wisdom; and to test the longer-term effects at 2 and 8 months respectively. Method: A waitlist randomised controlled trial (RCT) design was used. A total of 157 older adults were recruited, 82 of which was randomly assigned to 12 intervention groups to receive four 2-hour NT sessions using the ‘Tree of Life’ metaphor, to assess perceived wisdom at baseline (T1), at the end of treatment (T2), and at two (T3) and 8 months after treatment (T4). Results: Participants in the NT group showed significant improvements in the wisdom outcome measure [F(2.726, p = 0.041)]. As such, the results of latent growth curve models with time-invariant covariates for impact of NT on wisdom scores suggested significant effects two months after treatment (T3) with controlled the effects of age, gender and educational level [TML(11) = 17.306, p = 0.098, RMSEA = 0.079, CFI = 0.960]. Most improvements were sustained at 2- and 8-months post intervention. No adverse reaction was recorded in any of the cases mentioned at all study sites. Conclusion: The findings have significant theoretical contributions for professional social work practice to ground a new theory in understanding wisdom in older adulthood, develop a new clinical practice that appreciate and celebrate life wisdom, and a practice guide to be disseminated among health and social care practitioners.

